# Converting from the Montreal Cognitive Assessment to the Mini-Mental State Examination-2

**DOI:** 10.1371/journal.pone.0254055

**Published:** 2021-07-08

**Authors:** Hwabeen Yang, Daehyuk Yim, Moon Ho Park

**Affiliations:** Department of Neurology, Korea University Ansan Hospital, Ansan, Korea; Cardiff University, UNITED KINGDOM

## Abstract

**Objective:**

The Montreal Cognitive Assessment (MoCA) and Mini-Mental State Examination-2 (MMSE-2) are useful psychometric tests for cognitive screening. Many clinicians want to predict the MMSE-2 score based on the MoCA score. To facilitate the transition from the MoCA to the MMSE-2, this study developed a conversion method.

**Methods:**

This study retrospectively examined the relationship between the MoCA and MMSE-2. Overall, 303 participants were evaluated. We produced a conversion table using the equipercentile equating method with log-linear smoothing. Then, we evaluated the reliability and accuracy of this algorithm to convert the MoCA to the MMSE-2.

**Results:**

MoCA scores were converted to MMSE-2 scores according to a conversion table that achieved a reliability of 0.961 (intraclass correlation). The accuracy of this algorithm was 84.5% within 3 points difference from the raw score.

**Conclusions:**

This study reports a reliable and easy conversion algorithm for transforming MoCA scores into converted MMSE-2 scores. This method will greatly enhance the utility of existing cognitive data in clinical and research settings.

## Introduction

The Mini-Mental State Examination (MMSE) is the most widely used cognitive screening test. In 2010, the MMSE-Second Edition, a revised version of the MMSE, was introduced. Importantly, MMSE-Second Edition: Standard version (MMSE-2) scores can be switched from MMSE scores without any score conversion [1]. Most clinical studies for dementia medication have used the MMSE score or changes in this score to determine the severity of dementia or effectiveness of treatments [2, 3]. Verifying MMSE scores in clinical practice is very important because many dementia practice guidelines refer to MMSE scores as a standardized tool for cognitive testing [4, 5]. However, under copyright restrictions, the MMSE can no longer be used, and the MMSE-2 must be purchased, potentially limiting its routine use in clinical and research settings [6].

The Montreal Cognitive Assessment (MoCA) is another cognitive screening test that has higher sensitivity than the MMSE for detecting early-stage cognitive decline [7]. The use of the MoCA is increasing for cognitive screening because it can be obtained free of charge and it has specific clinical characteristics.

Unfortunately, clinical trials vary in their use of these two scales, which makes comparisons between studies and meta-analyses difficult because the direct comparison of scores between the MMSE and MoCA is complicated. In addition, many clinicians want to predict the MMSE score based on the MoCA score. Previous studies have attempted to develop MoCA to MMSE conversion algorithms or equivalency tables [8–14]. These conversion methods have been validated in other languages [15, 16]. However, to the best of our knowledge, no study has converted the MoCA to the MMSE-2. Therefore, this study attempted to derive a conversion table from the MoCA to the MMSE-2 to develop a simple and reliable conversion algorithm for the two scales and to compare the new conversion algorithm with those described in previous studies.

## Methods

### Participants

This was a retrospective observational study of subjects who visited a memory clinic at a university hospital in the Republic of Korea and who were referred for neuropsychological screening. Overall, 303 participants who had visited the hospital from April 2020 to February 2021 were evaluated. The participants included 95 subjects with dementia, 172 subjects with mild cognitive impairment (MCI), and 36 cognitively unimpaired (CU) subjects.

MMSE data were referenced in this study because there is little information on the relationship between the MoCA and MMSE-2. A previous study reported that the converted score of the MMSE and the raw (original, actual, observed) score of the MMSE had an intraclass correlation coefficient (ICC) of 0.85 [17]. A sample size requirement of 31 subjects was calculated using Walter’s method at a confidence interval of 0.95, a confidence interval width within ± 0.1, two raters per subject, and a given ICC of 0.85 [18]. The current study met and exceeded the minimal requirements of these sample sizes for each cognitive subgroup.

Subjects with dementia met the criteria for a major neurocognitive disorder proposed by the Diagnostic and Statistical Manual of Mental Disorders (DSM‐5), American Psychiatric Association [19]. Subjects with MCI were diagnosed according to the criteria proposed by the International Working Group on MCI [20]. In this study, CU subjects did not meet the criteria for MCI or dementia but were recruited and assessed in a manner identical to that used for those with MCI and dementia [21]. CU subjects were functionally independent.

Demographic data, including age and sex, and information regarding years of education were collected. All participants underwent a comprehensive evaluation consisting of a detailed medical history, neurological examinations, and a neuropsychological evaluation. In addition, laboratory tests were used to confirm there were no other causes for dementia or cognitive impairment. Magnetic resonance imaging was performed on all patients. All participants underwent the MMSE-2 followed by the MoCA on the same day. The results of the MoCA and MMSE-2 were not available during the consensus diagnosis.

The study protocol was reviewed and approved by the Institutional Review Board of Korea University Ansan Hospital (2021AS0066), and informed consent was not necessary because of the retrospective design of the study and the de-identified nature of the data.

### MoCA and MMSE-2

The MoCA is the most widely used screening test and was developed as a brief screening test for MCI and the early stages of dementia [7, 22]. This test evaluates visuospatial (5 points), naming (3 points), attention (6 points), language (3 points), abstract (2 points), memory (5 points), and orientation (6 points) abilities. Possible scores range from 0 to 30 points, where higher scores indicate better cognitive function. This study used the Korean version of the MoCA [22].

The MMSE-2 is the most commonly used test to screen for cognitive impairment and has been the most extensively used in clinical and research settings due to its practicality [1]. The MMSE-2 has potential scores that range from 0 to 30 points, where higher scores indicate better cognitive function. The MMSE-2, similar to the MMSE, examines the following six cognitive domains: orientation in time (5 points), orientation in place (5 points), memory registration (3 points), memory recall (3 points), attention and calculation (5 points), and language and other functions (8 points). This study used the Korean version of MMSE-2: Standard Version, Blue Form [23].

### Statistical analysis

Data are expressed as the means (standard deviations) for continuous variables and as percentages for categorical variables. Demographic and clinical characteristics were evaluated with chi-squared tests for differences between proportions, and the Kruskal–Wallis test was used to test for differences between continuous variables after performing Levene’s test for equality of variance. Bonferroni correction was used for post-hoc comparisons.

The overall agreement between the MoCA and MMSE-2 was assessed using Pearson’s correlation coefficient (*r*). For comparisons among correlations for cognitive subgroups, coefficients were converted and compared with Fisher’s Z-transformation. In addition, the concordance correlation coefficient (CCC) was also calculated. The CCC is a more conservative measure of agreement without the issue of linear versus nonlinear association, which measures agreement by assessing how well the relationship between the measurements is represented by a line through the origin at an angle of 45 degrees. The CCC values were interpreted as poor (CCC < 0.90), moderate (CCC = 0.90–0.95), substantial (CCC = 0.95–0.99), or almost perfect (CCC > 0.99) [24].

The equipercentile equating method with log-linear smoothing was used to estimate scores from the MoCA to the MMSE-2. This equating method matched the MoCA score and the raw score of the MMSE-2 based on their respective percentile ranks after smoothing the corresponding distribution. A comprehensive explanation of equipercentile equating has been described previously [25]; in summary, scores from two different measures are considered equivalent if their corresponding percentile ranks are equal. The strength of this method is that the equated scores always fall within the range of possible scores; a limitation is that this method can lead to an irregular distribution of scores. A log-linear transformation of the raw value of each score before equipercentile equating is required to smooth the raw scores and to create a normal distribution without irregularities that are attributable to sampling. Log-linear transformation enhances the equating accuracy. Based on results from the equipercentile equating analysis, MMSE-2 converted scores with equating were made. For equipercentile equating analyses, although MoCA and MMSE-2 scores are continuous, they are integers without decimal points; thus, all estimating scores were made to the nearest integer, which restricted the range of the score to between 0 to 30.

We evaluated the converting method in this study using the ICC to measure the agreement between the raw and converted MMSE-2 scores according to cognitive subgroups. The ICC values were interpreted as poor (ICC < 0.40), fair (ICC  =  0.40–0.59), good (ICC  =  0.60–0.74), or excellent (ICC  =  0.75–1.0) [26]. Moreover, the agreement between the raw and converted MMSE-2 score was determined by examining a Bland-Altman plot [27], with a limit of agreement (LOA) ± 1.96 standard deviations from the mean difference. The 95% LOA between the raw MMSE-2 score and the converted score expressed the degree of error proportional to the mean of the measurement units. If the differences between the measurements tended to agree, the results were close to zero. These plots showed the difference between each pair of measurements on the y-axis against the mean of each pair of measurements on the x-axis. Bias was assessed using a linear regression analysis.

Finally, we evaluated the accuracy of the conversion algorithm with a percentage of converted scores within 0, ± 1–2, and ± 3 points of error, where an error was defined as the difference between the raw of MMSE-2 score and the converted score. In addition, the accuracy of previous methods [8–10] for converting from MoCA to MMSE scores have been evaluated and compared with the those of converting from MoCA to MMSE-2 scores because it is possible to switch from the MMSE to the MMSE-2 without any change in their scores [1].

Analyses were performed using SPSS for Windows, version 20.0 (IBM Corporation, Armonk, NY, USA) and R 4.0.2 software with its appropriate packages (The R Foundation for Statistical Computing, Vienna, Austria). Statistical tests were two-tailed, and α was set at <0.05.

## Results

The demographic and clinical data of the study population are summarized in Table 1. The mean age of all participants was 70.52 (10.74) years, and the mean duration of education was 7.78 (4.86) years. Approximately 57% of participants were women. There were statistical differences in age, sex, and education among the cognitive subgroups. In addition, the mean raw MMSE-2 scores were higher than the mean MoCA scores. As expected, each mean MMSE-2 and MoCA raw score of was statistically significantly different according to cognitive subgroups.

**Table 1:** Demographic data and MoCA and MMSE-2 scores and their correlations.

	Total	Dementia	MCI	CU	P^*^
	(n = 303)	(n = 95)	(n = 172)	(n = 36)	
**Demographics**					
Age, years	70.52 (10.74)	75.25 (9.31)	69.08 (10.40)	64.94 (11.49)	<0.001^a^
Sex, female	173 (57.1%)	52 (54.7%)	100 (58.1%)	21 (58.3%)	0.854^b^
Education, year	7.78 (4.86)	6.74 (4.98)	7.90 (4.76)	9.96 (4.31)	0.002^a^
**Cognitive screening test**					
MMSE-2	21.37 (7.02)	14.08 (6.39)	23.96 (4.16)	28.19 (2.35)	<0.001^a^
	[23 (17–27)]	[14 (9–19)]	[25 (22.25–27)]	[29 (27.25–30)]	
MoCA	14.91 (7.41)	7.62 (5.49)	17.10 (5.25)	23.67 (3.41)	<0.001^a^
	[16 (10–21)]	[7 (3–11)]	[18 (14–21)]	[24 (22–25)]	
**MoCA-MMSE-2 correlation**					
Pearson’s *r*	0.916**	0.864**	0.849**	0.681**	
CCC	0.652	0.535	0.402	0.286	
	[0.607–0.693]	[0.441–0.618]	[0.568–0.688]	[0.144–0.416]	

Note. Values are presented as the means (standard deviations) or numbers (%). The MMSE-2 and MoCA scores of are additionally presented as the median (interquartile range) with square brackets. The correlation coefficients are presented as coefficient values or its values [95% confidence interval]. P^*^ values were compared among cognitive subgroups.

^a^Kruskal-Wallis test.

^b^Chi-squared test. P^**^ < 0.01.

Abbreviations: MCI, mild cognitive impairment; CU, cognitively unimpaired; MMSE, Mini-Mental State Examination; MoCA, Montreal Cognitive Assessment; CCC, concordance correlation coefficient.

### Agreement of the two scales (MoCA and MMSE-2)

For all participants, Pearson’s correlation coefficient (*r*) for the raw MMSE-2 score and MoCA score was 0.916 (*P*<0.01) (Table 1), which indicated strong agreement [28].

Among the cognitive subgroups, there were no statistical differences in Pearson’s correlation coefficient for the MMSE-2 and MoCA between subjects with dementia and MCI (*z* = 0.435), between those with dementia and CU individuals (*z* = 2.355), and between those with MCI and CU individuals (*z* = 2.215) (all *P*>0.05 after Bonferroni correction).

In addition, the CCC between the raw MMSE-2 score and the MoCA score was 0.652 (95% confidence interval [CI], 0.607–0.693) for all participants, indicating poor agreement [24]. Table 1 shows each CCC according to cognitive subgroup.

### Conversion table

**Fig 1:**
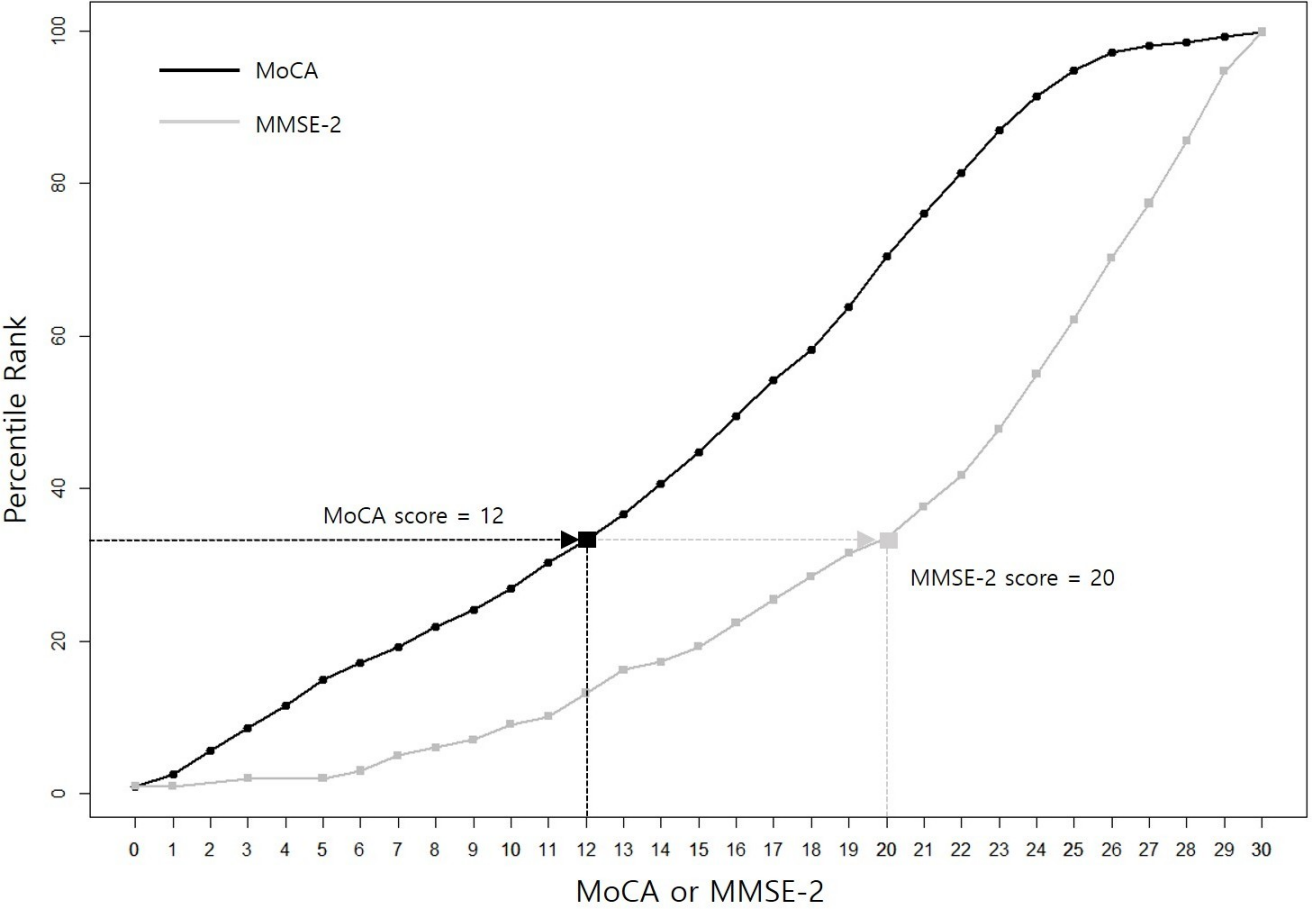
Equipercentile equating of the MoCA and MMSE-2. Equipercentile equating of the MoCA (black color) and MMSE-2 (gray color) corresponding to test scores and percentiles allows conversion of MoCA scores to MMSE-2 scores.

The plot of equipercentile equivalents of MoCA and MMSE-2 scores is presented in Fig 1. For example, an MoCA score of 12 is equivalent to an MMSE-2 score of 20, with both of these scores falling at approximately the same percentile rank. Table 2 shows MoCA scores and their respective equivalents on the MMSE-2.

**Table 2:** Equipercentile equating table for potential conversion of MoCA scores to MMSE-2 scores.

MoCA score	Equivalent MMSE-2 score
0	0
1	2
2	4
3	7
4	10
5	13
6	14
7	16
8	17
9	18
10	19
11	20
12	20
13	21
14	22
15	22
16	23
17	24
18	25
19	26
20	26
21	27
22	27
23	28
24	28
25	29
26	29
27	30
28	30
29	30
30	30

Abbreviations: MoCA, Montreal Cognitive Assessment; MMSE, Mini-Mental State Examination.

### Reliability of raw and converted MMSE-2 scores

For all participants, the analysis of the reliability of the raw and converted MMSE-2 scores showed excellent intrarater reliability with an ICC_(2,1)_ = 0.961 (Table 3). Moreover, according to the cognitive subgroups, the ICC_(2,1)_ between these scores also had excellent reliability.

**Table 3:** Analysis of reliability between the converted and raw MMSE-2 scores according to cognitive subgroups.

Total	Dementia	MCI	CU
(n = 303)	(n = 95)	(n = 172)	(n = 36)
0.961	0.924	0.919	0.849
(0.952–0.969)	(0.886–0.946)	(0.890–0.940)	(0.703–0.923)

Note. Values are presented as intraclass correlation coefficients (95% confidence intervals).

Abbreviations: MCI, mild cognitive impairment; CU, cognitively unimpaired; MMSE, Mini-Mental State Examination.

**Fig 2:**
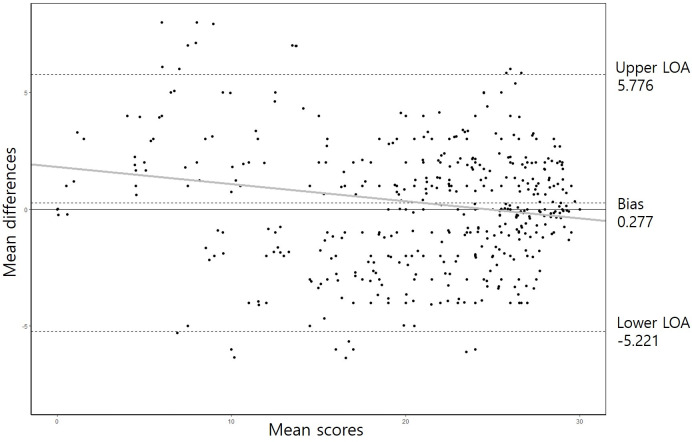
A Bland-Altman plot of the difference in the raw and converted MMSE-2 scores. The solid line indicates the reference (no mean difference), the middle-dotted line is the mean difference, and the upper- and lower-dotted lines are the limits of agreement representing ±1.96 standard deviations (SDs) from the mean difference within which 95% of the differences between the two scores are expected to fall. The gray solid lines are the fitted regression lines, indicating a significant linear trend.

A Bland-Altman plot showed that the mean difference between the raw and converted MMSE-2 scores was 0.277 with an upper and a lower LOA of 5.776 and −5.221, respectively. The scores of 286 participants (94.4%) fell within or on the 95% LOA, with a reasonably even distribution across the mean scores. However, there was an indication of bias according to the regression coefficient (*y* = −0.073×*x*+1.8, *P*<0.05) (Fig 2).

### Accuracy of the converted MMSE-2

This study showed that 70.0% of the converted MMSE-2 scores were within ± 1–2 points of the raw MMSE-2 scores, and 84.5% of the converted score of MMSE-2 were within ± 3 points of the raw score of MMSE-2 (Table 4).

**Table 4:** Accuracy of the converted MMSE-2 scores against the clinically administered raw MMSE-2 scores.

Methods	*Difference between the raw score and the converted score of MMSE-2		
	(0)	(±1~2)	(±3)
Current study	16.8%	70.0%	84.5%
Roalf et al [8]	19.1%	68.6%	83.5%
van Steenoven et al [9]	15.2%	59.1%	72.6%
Trzepacz et al [10]	17.8%	66.3%	82.5%

Note: *These differences (accuracies) in four methods were evaluated with this study participants.

Abbreviations: MMSE, Mini-Mental State Examination.

## Discussion

The purpose of this study was to facilitate the transition from the MoCA to the MMSE-2. This study reports a simple and reliable method for converting the MoCA to the MMSE-2. Using a conversion table, MoCA scores can be expressed in MMSE-2 terms. This conversion algorithm provides a straightforward way of comparing the MoCA with the MMSE-2, allowing for continuity of cognitive tracking in clinical settings and comparability of data between longitudinal studies. To the best of our knowledge, this is the first study to examine conversion from the MoCA to the MMSE-2.

To construct a reliable algorithm to convert scores between the MoCA and MMSE-2, we initially evaluated the relationship between these two scales. It is often assumed that because the MoCA and MMSE-2 measure the same general construct of cognition that they are easily interchangeable. However, regarding agreement properties, Pearson’s correlation coefficient for the MoCA and MMSE-2 indicated strong agreement in this study, but the CCC indicated poor agreement because the latter presumably evaluates components of the degree of variation and degree of location or scale shift [24]. For psychometric properties, the MoCA and MMSE-2 emphasize different aspects of cognition: the MoCA examines more cognitive domains than the MMSE-2, including executive functions and attention [7]. Based on these agreement and psychometric properties, this study evaluated the relationship between the MoCA and MMSE-2 to construct a conversion table using an equipercentile equating method rather than simple linear analyses. In addition, this study chose a conversion table with reliability and intuitive and convenient usage in clinical practice.

Equipercentile equating methods have been widely used in previous studies [8–13] because these methods enable direct and easy comparison of scores. Previous studies did not evaluate the entire range of MoCA scores (0–30 points) [11, 12]; however, this study introduced a conversion table including MMSE-2 scores corresponding to all possible MoCA scores.

Reliability and agreement are other considerations in this conversion algorithm. This study showed an excellent ICC between the raw and converted MMSE-2 scores. Furthermore, this excellent ICC was maintained among cognitive subgroups and among all subjects. Our conversion rule compared very favorably with those described in previous studies, demonstrating improved agreement. However, the Bland-Altman plot showed systemic bias in the agreement between the raw and converted scores of the two tests. The conversion table showed a negative correlation between the mean and the difference. Therefore, considering these characteristics, correlations should be evaluated carefully when using this algorithm.

To facilitate the transition from the MoCA to the MMSE-2, performance on the MoCA must be translated into the MMSE-2 within an acceptable margin of error. Thus, this study evaluated the accuracy of the algorithm used for the conversion of MMSE-2 scores. In this study, the Bland-Altman analyses showed the ranges of the upper and lower LOAs were within approximately ±5 points of difference between the raw and converted MMSE-2 scores. However, this study chose ± 0–3 points of difference. In previous studies, the reliable change score, which is an individual’s change in test performance that refers to real changes in underlying cognitive abilities and not to chance trends, was reported to be 3 points [23] or 4 points [1] for the MMSE-2 and 2–4 points [29], 3–4 points [30], or 3.3 points [31] for the MMSE. With reference to these reliable change scores, this study evaluated the accuracy of the converted MMSE-2 score by the difference from the raw score and within 0, ± 1–2, and ± 3 points. The conversion algorithm used in this study had an accuracy of 16.8% with no difference (perfect matched score) and an accuracy of 84.5% within 3 points of difference between the raw and converted MMSE-2 scores. Using the previously suggested methods [8–10], we obtained an accuracy of 72.6–83.5% within 3 points of difference between the raw and converted MMSE-2 scores. This suggests that the conversion algorithm used in this study is comparable with the previously suggested methods.

This study had some limitations. First, it was subject to all of the limitations inherent to the use of a retrospective study design. In addition, there may be some degree of selection bias in this retrospective study. A prospective study is therefore warranted to validate our results. Second, the data in this study were collected from patients in the Korean population, and the generalizability of score mapping in other conditions such as Parkinson’s disease or stroke remains to be tested; thus, the relationship between MoCA and MMSE-2 scores may differ between other demographic or clinical conditions. Participants with subjective cognitive decline might have been recruited as CU subjects. No dementia subtypes or MCI subtypes were specifically examined. Third, most participants with higher MoCA scores (26 or higher MoCA scores) had near the maximum MMSE-2 scores, and most participants with lower MMSE-2 scores (5 or lower MMSE-2 scores) had near the minimum MoCA scores because the MoCA is generally more difficult than the MMSE-2. Participants with lower or higher MoCA scores require further validation because the conversion scales utilized a narrow distribution of MoCA or MMSE-2 scores. Fourth, the order of administration of the MMSE and MoCA was not randomized to minimize the effect of learning one test prior to taking the other. Furthermore, because the tests were administered in a specific language, the generalizability of the score conversion to other languages needs to be explored further. Fifth, this study evaluated only basic information, including age, sex, and education level, as the real primary clinical field. However, other comorbidities and laboratory parameters can affect cognitive function [32]. A more detailed screening evaluation using these variables should be performed.

In conclusion, this study validated an algorithm to convert MoCA scores to MMSE-2 scores to allow comparison of data from these two cognitive screening tests. The findings of this study should serve as a useful reference for clinicians to continue clinical care using the MMSE-2 in subjects who were previously administered the MoCA. This will greatly enhance the utility of existing research data and facilitate greater collaboration and shared analyses, leading to more robust research findings.

## Supporting information

S1 Data (CSV)
