# Supplementary material for: Converting from the Montreal Cognitive Assessment to the Mini-Mental State Examination-2
Source: PLoS One. 2021 Jul 8;16(7):e0254055. doi: 10.1371/journal.pone.0254055 (PMC8266092; doi:10.1371/journal.pone.0254055)
Supplement: Peer review history

# Peer review: Decision Letter 0

**DOI:** 10.1371/journal.pone.0254055.r001

**Authors:** Antony Bayer

13 May 2021

PONE-D-21-11110

Converting from Montreal Cognitive Assessment to Mini-Mental State Examination-2

PLOS ONE

Dear Dr. PARK,

Thank you for submitting your manuscript to PLOS ONE. After careful consideration, we feel that it has merit but does not fully meet PLOS ONE’s publication criteria as it currently stands. Therefore, we invite you to submit a revised version of the manuscript that addresses the points raised during the review process that are detailed below. Particular issues seem to be the origin of the study population, the order of testing, and the validity of the conversion across the full range of scores (0-30).

Please submit your revised manuscript by Jun 26 2021 11:59PM. If you will need more time than this to complete your revisions, please reply to this message or contact the journal office at plosone@plos.org. When you're ready to submit your revision, log on to https://www.editorialmanager.com/pone/ and select the 'Submissions Needing Revision' folder to locate your manuscript file.

Please include the following items when submitting your revised manuscript:

- A rebuttal letter that responds to each point raised by the academic editor and reviewer(s). You should upload this letter as a separate file labeled 'Response to Reviewers'.
- A marked-up copy of your manuscript that highlights changes made to the original version. You should upload this as a separate file labeled 'Revised Manuscript with Track Changes'.
- An unmarked version of your revised paper without tracked changes. You should upload this as a separate file labeled 'Manuscript'.

If you would like to make changes to your financial disclosure, please include your updated statement in your cover letter. Guidelines for resubmitting your figure files are available below the reviewer comments at the end of this letter.

If applicable, we recommend that you deposit your laboratory protocols in protocols.io to enhance the reproducibility of your results. Protocols.io assigns your protocol its own identifier (DOI) so that it can be cited independently in the future. For instructions see: http://journals.plos.org/plosone/s/submission-guidelines#loc-laboratory-protocols. Additionally, PLOS ONE offers an option for publishing peer-reviewed Lab Protocol articles, which describe protocols hosted on protocols.io. Read more information on sharing protocols at https://plos.org/protocols?utm_medium=editorial-email&utm_source=authorletters&utm_campaign=protocols.

We look forward to receiving your revised manuscript.

Kind regards,

Antony Bayer

Academic Editor

PLOS ONE

Journal Requirements:

When submitting your revision, we need you to address these additional requirements.

1. Please ensure that your manuscript meets PLOS ONE's style requirements, including those for file naming. The PLOS ONE style templates can be found at

https://journals.plos.org/plosone/s/file?id=wjVg/PLOSOne_formatting_sample_main_body.pdf and

https://journals.plos.org/plosone/s/file?id=ba62/PLOSOne_formatting_sample_title_authors_affiliations.pdf

2. Thank you for including your ethics statement:

"The study protocol was reviewed and approved by the Institutional Review Board of Korea University Ansan Hospital (2021AS0066) and informed consent was not necessary because of the study’s retrospective design and the de-identified nature of the data."

a) Please provide additional details regarding participant consent. In the ethics statement in the Methods and online submission information, please ensure that you have specified what type you obtained (for instance, written or verbal, and if verbal, how it was documented and witnessed). If your study included minors, state whether you obtained consent from parents or guardians. If the need for consent was waived by the ethics committee, please include this information.

Once you have amended this/these statement(s) in the Methods section of the manuscript, please add the same text to the “Ethics Statement” field of the submission form (via “Edit Submission”).

For additional information about PLOS ONE ethical requirements for human subjects research, please refer to http://journals.plos.org/plosone/s/submission-guidelines#loc-human-subjects-research.

3. Please include captions for your Supporting Information files at the end of your manuscript, and update any in-text citations to match accordingly. Please see our Supporting Information guidelines for more information: http://journals.plos.org/plosone/s/supporting-information.

[Note: HTML markup is below. Please do not edit.]

Reviewers' comments:

Reviewer's Responses to Questions

**Comments to the Author**

1. Is the manuscript technically sound, and do the data support the conclusions?

The manuscript must describe a technically sound piece of scientific research with data that supports the conclusions. Experiments must have been conducted rigorously, with appropriate controls, replication, and sample sizes. The conclusions must be drawn appropriately based on the data presented. 

Reviewer #1: Yes

Reviewer #2: Yes

Reviewer #3: Yes

**********

2. Has the statistical analysis been performed appropriately and rigorously? 

Reviewer #1: I Don't Know

Reviewer #2: Yes

Reviewer #3: Yes

**********

3. Have the authors made all data underlying the findings in their manuscript fully available?

The PLOS Data policy requires authors to make all data underlying the findings described in their manuscript fully available without restriction, with rare exception (please refer to the Data Availability Statement in the manuscript PDF file). The data should be provided as part of the manuscript or its supporting information, or deposited to a public repository. For example, in addition to summary statistics, the data points behind means, medians and variance measures should be available. If there are restrictions on publicly sharing data—e.g. participant privacy or use of data from a third party—those must be specified.

Reviewer #1: Yes

Reviewer #2: Yes

Reviewer #3: Yes

**********

4. Is the manuscript presented in an intelligible fashion and written in standard English?

PLOS ONE does not copyedit accepted manuscripts, so the language in submitted articles must be clear, correct, and unambiguous. Any typographical or grammatical errors should be corrected at revision, so please note any specific errors here.

Reviewer #1: Yes

Reviewer #2: No

Reviewer #3: Yes

**********

5. Review Comments to the Author

Please use the space provided to explain your answers to the questions above. You may also include additional comments for the author, including concerns about dual publication, research ethics, or publication ethics. (Please upload your review as an attachment if it exceeds 20,000 characters)

Reviewer #1: Simple methods to convert test scores from commonly administered cognitive screening instruments (CSIs) to approximate MMSE scores is of recognised clinical utility. There are various methods for doing this, including calculation of linear regression equations and, as in this paper, deriving a conversion table of equivalent scores from equipercentile equating with log-linear smoothing. A potential problem with the latter is that it includes all those MMSE items which are recognized to be easy and which are of little value in patient assessment.

Queries to address:

Introduction

P3: “Previous studies have attempted to develop MoCA to MMSE

conversion algorithms or equivalent tables”. The authors might also include here Int J Geriatr Psychiatry 2017;32:351-2.

Methods

P4-5: 303 study participants who attended a clinic. Was this all who attended, or were some patients unable/unwilling to complete bot MoCA and MMSE-2, or other aspects of the diagnostic assessment?

P4: “36 subjects with being cognitively unimpaired (CU).”. Were these healthy controls, in which case this is an experimental study, or patients referred to the clinic with subjective memory complaints, in which case you have a pragmatic study? This sentence in the Discussion (P16) “Participants with subjective cognitive decline might have been recruited as subjects with CU.” suggests the former. This point has important implications for the potential generalisability or transferability of the equipercentile equating table.

P5: “All participants underwent the MoCA and MMSE-2 examinations on the same day”. Were these performed in a counterbalanced order to avoid bias? Answered in the Discussion at P16.

Results:

P13: Figure 2 legend implies 3 solid and 2 dotted lines, whereas Figure 2 as presented on P29 has 2 solid and 3 dotted lines. Interpretation not clear.

Reviewer #2: PONE-D-21-11110

Converting from Montreal Cognitive Assessment to Mini-Mental State Examination-2

The author add to a growing literature of cross-walks between cognitive screening tests in aging/dementia. The authors implement previously validated methods to compare the MoCA and MMSE-2 in a moderately sized aging sample that included cognitive normal as well as MCI and AD patients. The author report that the MoCA and MMSE-2 can reliably be converted, and, in general, show that the conversion is similar to previous work converting MoCA to MMSE. The authors should address a few concerns to improve the current state the manuscript:

General: The manuscript would benefit from thorough copy-editing for grammar.

Methods/Results:

-The authors should state whether the order of test was consistent. Was MMSE-2 always given before the MoCA?

-In the description of the MoCA and MMSE-2 the authors state the MoCA is ‘the most widely used screening test for cognitive dysfunction” and then state that the MMSE-2 is ‘the most commonly used test for the screening of cognitive impairment’: I find it rather difficult to discern the difference between these two claims. Can the authors provide more detail about how these differ?

-In my opinion there is no need to include the explanation of the meanings of commonly used stats in terms of relative strength (e.g. Pearson r).

-The authors need to provide the robust range of MMSE-2 and MoCA scores in each of the samples. That is, what are the minimum and maximum scores? This is relevant for understanding equipercentile equating and the LOA. It is likely that most of the lower end of the scales (particularly for the MMSE-2) is not observed, thus making scores at the lower range less stable b/c more interpolation is needed in the equating approach. There is not much to overcome this, but should be discussed as a likely contributor to less concordant scores at the lower end of the scales.

-What is the benefit or the point in comparing the Pearson r-values between MMSE-2 and MoCA to other studies? Other important factor such as sample size, age, education, etc. cannot be accounted for and could explain these differences. This does not seem to be thoroughly discussed and as such make me question why this analysis was performed and what additional information is gained from this analysis.

-The authors should provide more clarity on Table 4 as the methods are not clear. I believe that the authors are using previously published conversion tables on their sample. It should be made clear that other cross-walks are being applied to their sample and that the author are did not recalculate data from the originals sources.

Reviewer #3: In this manuscript, the authors present a conversion table from the MoCA to the MMSE-2 using the equipercentile equating method with log-linear smoothing.

The manuscript is well-written and the statistical analyses have been properly performed.

I have some minor comments:

- the sample size is relatively small, especially regarding patients with dementia (n=94). The authors present the conversion table for all MoCA scores (including MoCA = 0). It would be important to mention how many patients had a MoCA 0-5, 5-10, etc. in the sample (which seems low based on figure 2) and discuss whether the conversion is valid at these low scores given the low sample size

- A mean MoCA of 17 seems low for patients with MCI. I would expect that some patients with MoCA<15 in this group probably have a functional impairment suggestive of dementia.

- authors could provide more detail regarding the cause of cognitive impairment of patients included in the sample. For instance, it is known that the conversion between two cognitive tests is slightly different in patients with vascular dementia (dysexecutive profile) compared with patients with Alzheimer's disease (amnestic profile).

- In Table 1, I would refer to the MMSE-2 and MoCA as cognitive screening tests rather than neuropsychological tests.

Altogether, my recommendation is to accept the manuscript pending minor revisions.

**********

6. PLOS authors have the option to publish the peer review history of their article (what does this mean?). If published, this will include your full peer review and any attached files.

If you choose “no”, your identity will remain anonymous but your review may still be made public.

**Do you want your identity to be public for this peer review?** For information about this choice, including consent withdrawal, please see our Privacy Policy.

Reviewer #1: **Yes: **Andrew J Larner

Reviewer #2: **Yes: **David R. Roalf

Reviewer #3: **Yes: **David Bergeron

[NOTE: If reviewer comments were submitted as an attachment file, they will be attached to this email and accessible via the submission site. Please log into your account, locate the manuscript record, and check for the action link "View Attachments". If this link does not appear, there are no attachment files.]

While revising your submission, please upload your figure files to the Preflight Analysis and Conversion Engine (PACE) digital diagnostic tool, https://pacev2.apexcovantage.com/. PACE helps ensure that figures meet PLOS requirements. To use PACE, you must first register as a user. Registration is free. Then, login and navigate to the UPLOAD tab, where you will find detailed instructions on how to use the tool. If you encounter any issues or have any questions when using PACE, please email PLOS at figures@plos.org. Please note that Supporting Information files do not need this step.

# Author comment: Author response to Decision Letter 0

**DOI:** 10.1371/journal.pone.0254055.r002

3 Jun 2021

Reviewer #1: Simple methods to convert test scores from commonly administered cognitive screening instruments (CSIs) to approximate MMSE scores is of recognised clinical utility. There are various methods for doing this, including calculation of linear regression equations and, as in this paper, deriving a conversion table of equivalent scores from equipercentile equating with log-linear smoothing. A potential problem with the latter is that it includes all those MMSE items which are recognized to be easy and which are of little value in patient assessment.

Queries to address:

Introduction

P3: “Previous studies have attempted to develop MoCA to MMSE

conversion algorithms or equivalent tables”. The authors might also include here Int J Geriatr Psychiatry 2017;32:351-2.

→ Thank you for this comment. As the reviewer’s comments, we added the reference: Int J Geriatr Psychiatry 2017;32:351-2.

14. Larner AJ. Converting cognitive screening instrument test scores to MMSE scores: regression equations. Int J Geriatr Psychiatry 2017;32(3):351-2. doi: 10.1002/gps.4622 PMID: 28170137

Methods

P4-5: 303 study participants who attended a clinic. Was this all who attended, or were some patients unable/unwilling to complete bot MoCA and MMSE-2, or other aspects of the diagnostic assessment?

→ We are grateful for this valuable comment. The basic screening tests for all participants visiting the memory clinic are the MoCA and the MMSE-2. The MoCA and the MMSE-2 tests were evaluated all subjects (n=303) who visited the memory clinic during the study period.

P4: “36 subjects with being cognitively unimpaired (CU).”. Were these healthy controls, in which case this is an experimental study, or patients referred to the clinic with subjective memory complaints, in which case you have a pragmatic study? This sentence in the Discussion (P16) “Participants with subjective cognitive decline might have been recruited as subjects with CU.” suggests the former. This point has important implications for the potential generalisability or transferability of the equipercentile equating table.

→ We are grateful for this insightful comment. There are various participants/patients who are referred to the memory clinic. The patients with MCI or dementia are commonly referred to, but the subjects who want to be evaluated for only cognitive screening test, the subjects with subjective memory complaints, or the subject who want pre-evaluation with high risk of cognitive decline, etc.. Many of them currently have normal cognitive function, but we fully agree that they are clinically heterogenous. However, in this study, since there were not many participants with normal cognitive function, these subjects were combined as the CU (cognitively normal) group. We fully agree with the weakness pointed out, and have described these limitations in the discussion section.

P5: “All participants underwent the MoCA and MMSE-2 examinations on the same day”. Were these performed in a counterbalanced order to avoid bias? Answered in the Discussion at P16.

→ We are grateful for this kind comment. Unfortunately, this is a retrospective analysis and we didn’t control the order of two tests’ performance. As the reviewer’s comments, there is a bias of unperforming in a counterbalanced order of two tests. Thus, we mentioned it in discussion section- limitation part at P17.

Results:

P13: Figure 2 legend implies 3 solid and 2 dotted lines, whereas Figure 2 as presented on P29 has 2 solid and 3 dotted lines. Interpretation not clear.

→ We are grateful for this kind comment. We are very sorry that there is a mistake in figure 2 legend. We revised and re-wrote the figure 2 legend as the reviewer’s comments as below:

“The solid line indicates the reference (no mean difference), the middle-dotted line is the mean difference, and the upper- and lower-dotted lines are the limits of agreement…”

Reviewer #2: PONE-D-21-11110

Converting from Montreal Cognitive Assessment to Mini-Mental State Examination-2

The author add to a growing literature of cross-walks between cognitive screening tests in aging/dementia. The authors implement previously validated methods to compare the MoCA and MMSE-2 in a moderately sized aging sample that included cognitive normal as well as MCI and AD patients. The author report that the MoCA and MMSE-2 can reliably be converted, and, in general, show that the conversion is similar to previous work converting MoCA to MMSE. The authors should address a few concerns to improve the current state the manuscript:

General: The manuscript would benefit from thorough copy-editing for grammar.

→ We are grateful for this valuable comment. We are very sorry for poor English grammar. The revision manuscript was edited by native speaker proofreading. In the revised manuscript, this proofreading was described in the acknowledgements.

“The authors appreciate Essay Review (https://essayreview.co.kr) for the English language editing.”

Methods/Results:

-The authors should state whether the order of test was consistent. Was MMSE-2 always given before the MoCA?

→ We are grateful for this insightful comment. As the reviwer’s comments, the order of tests (MoCA and MMSE-2) is important. Unfortunately, this study is a retrospective analysis and we didn’t perform in a counterbalanced order to avoid bias. In our memory clinic, we evaluated the MMSE-2 followed by the MoCA. We revised and re-wrote the methods section [P5, L10-11] as below:

“All participants underwent the MMSE-2 followed by the MoCA on the same day.”

-In the description of the MoCA and MMSE-2 the authors state the MoCA is ‘the most widely used screening test for cognitive dysfunction” and then state that the MMSE-2 is ‘the most commonly used test for the screening of cognitive impairment’: I find it rather difficult to discern the difference between these two claims. Can the authors provide more detail about how these differ?

→ We are grateful for this insightful comment. In the initial submitted manuscript, we described two tests shortly because these tests have been well known. However, as the reviewer’s comments, we revised and rewrote the methods section with more detailed description about two tests as below [P5, L18-21 ~ P6, L2-8]:

“The MoCA is the most widely used screening test and was developed as a brief screening test for MCI and the early stages of dementia. This test evaluates visuospatial (5 points), naming (3 points), attention (6 points), language (3 points), abstract (2 points), memory (5 points), and orientation (6 points) abilities... The MMSE-2 is the most commonly used test to screen for cognitive impairment and has been the most extensively used in clinical and research settings due to its practicality… The MMSE-2, similar to the MMSE, examines the following six cognitive domains: orientation in time (5 points), orientation in place (5 points), memory registration (3 points), memory recall (3 points), attention and calculation (5 points), and language and other functions (8 points)…”

-In my opinion there is no need to include the explanation of the meanings of commonly used stats in terms of relative strength (e.g. Pearson r).

→ We are grateful for this kind comment. In the initial submitted manuscript, we described the Pearson’s r with interpretation because there were the concordance correlation coefficient (CCC) and the intra-class correlaion with their interpretation. However, as the reviewer’s comments, the well-known stats is not needed its detailed description. Thus, we deleted the meanings of relative strength of the Pearson’s r as below [P6, L17-18]:

“The overall agreement between the MoCA and MMSE-2 was assessed using Pearson’s correlation coefficient (r). For comparisons among correlations for cognitive subgroups…”

-The authors need to provide the robust range of MMSE-2 and MoCA scores in each of the samples. That is, what are the minimum and maximum scores? This is relevant for understanding equipercentile equating and the LOA. It is likely that most of the lower end of the scales (particularly for the MMSE-2) is not observed, thus making scores at the lower range less stable b/c more interpolation is needed in the equating approach. There is not much to overcome this, but should be discussed as a likely contributor to less concordant scores at the lower end of the scales.

→ We are grateful for this insightful comment. With the reference to the reviewer’s comment, we added the mean and IQR of two tests’ scores (the minimum and maximum scores of two tests were 0 and 30, in this study). There is a difficult matter for equating of lower or upper end of the scales because the level of difficulty and cognitive domains of two scales are different. To overcome this difficulty, we evaluated the reliability (ICC) between the converted score and the raw score of MMSE-2 according to cognitive subgroups (dementia, MCI, and CU). Fortunately, in this study, the reliabilities of all subgroups can be interpretated as excellent (>0.75 in Table 3). The dementia subgroup relatively has lower ends of scale score and the CU subgroup has upper ends of scale score. Indirectly, we thought, the excellent reliabilities of both subgroups can be overcome the difficulty which is pointed out by the reviewer. We added these limitation of reliabilities for cognitive subgroups in discussion section (P16-17) and table 3.

“Third, most participants with higher MoCA scores (26 or higher MoCA scores) had near the maximum MMSE-2 scores, and most participants with lower MMSE-2 scores (5 or lower MMSE-2 scores) had near the minimum MoCA scores because the MoCA is generally more difficult than the MMSE-2. Participants with lower or higher MoCA scores require further validation because the conversion scales utilized a narrow distribution of MoCA or MMSE-2 scores.”

-What is the benefit or the point in comparing the Pearson r-values between MMSE-2 and MoCA to other studies? Other important factor such as sample size, age, education, etc. cannot be accounted for and could explain these differences. This does not seem to be thoroughly discussed and as such make me question why this analysis was performed and what additional information is gained from this analysis.

→ We are grateful for this valuable comment. As the reviewer’s comments, we agreed that it is no benefit in comparing the Pearson’s r-value to other studies. Thus, we deleted that comparing sentence. 

For the overall agreement, since Pearson’s r-value is used the most and has been evaluated in many previous studies, we also evaluated it for intuitive comparison. To convert from MoCA to MMSE-2, we thought that reasonable degree of overall agreement should be guaranteed. So we tested it by Pearson’s r-value and concordance correlation coefficient (CCC), which commonly evaluated agreement, but which also evaluated some different aspects. In this study, the Pearson’s r-value had high value, but the CCC had relative low value. The possible cause of this incongruity of two values was mentioned (psychomotor properties, etc.) in discussion section and we described that the equipercentile method would be useful rather than a simple linear conversion due to this incongruity of two scale values. 

-The authors should provide more clarity on Table 4 as the methods are not clear. I believe that the authors are using previously published conversion tables on their sample. It should be made clear that other cross-walks are being applied to their sample and that the author are did not recalculate data from the originals sources.

→ We are grateful for this kind comment. In table 4, all accuracies were evaluated with this study population to compare the results from four methods (current study + three previous methods). We revised and added the method description sentence in table legends as below [P14, L5-6]:

“*These differences (accuracies) in four methods were evaluated with this study participants.” 

Reviewer #3: In this manuscript, the authors present a conversion table from the MoCA to the MMSE-2 using the equipercentile equating method with log-linear smoothing.

The manuscript is well-written and the statistical analyses have been properly performed.

I have some minor comments:

- the sample size is relatively small, especially regarding patients with dementia (n=94). The authors present the conversion table for all MoCA scores (including MoCA = 0). It would be important to mention how many patients had a MoCA 0-5, 5-10, etc. in the sample (which seems low based on figure 2) and discuss whether the conversion is valid at these low scores given the low sample size

→ We are grateful for this kind comment. In this study, the number of participants with MoCA 0-4 scores are 42, that with MoCA 5-10 scores are 46, and that with MoCA 26-30 scores are 10. We absolutely agreed that the sample size is relatively small. Fortunately, the ICC between the converted MMSE-2 and the raw MMSE-2 had excellent reliability in not only dementia group but also CU group (Table 3). As the reviewer’s comments, we revised and added this limitation in discussion section [P17, L4-6] as below:

“Participants with lower or higher MoCA scores require further validation because the conversion scales utilized a narrow distribution of MoCA or MMSE-2 scores.”

- A mean MoCA of 17 seems low for patients with MCI. I would expect that some patients with MoCA<15 in this group probably have a functional impairment suggestive of dementia.

→ We are grateful for this valuable comment. We agreed that the MoCA score of MCI group is relatively low. We thought that this lower score is due to demographic characteristics of study participants: Korean people, lower education, and older age. In the validation study of K-MoCA (Ref 22. Kang et al. Korean J Clinical Psychology 2009), the mean score of MoCA is 18.39 (4.42) in all MCI subjects and that is 18.02 (5.62) in 65-79 years old (in this study, mean age of MCI group is about 70 years old). Thus, we wrote this limitation of selection bias should be overcome to re-tested with other demographics [P16, L20-21]:

“...thus, the relationship between MoCA and MMSE-2 scores may differ between other demographic or clinical conditions.”

- authors could provide more detail regarding the cause of cognitive impairment of patients included in the sample. For instance, it is known that the conversion between two cognitive tests is slightly different in patients with vascular dementia (dysexecutive profile) compared with patients with Alzheimer's disease (amnestic profile).

→ We are grateful for this insightful comment. We absolutely agreed that participants with vascular dementia and participants with Alzheimer’s disease had different cognitive profile and two scales’ score distribution. Because of the small sample size, the study protocol and IRB permission, and the relatively short MMSE-2 experience (in South Korea, MMSE-2 was officially published in April 2020), this study can’t evaluate the conversion table with various types of dementia (Alzheimer’s disease, vascular dementia, Parkinson’s disease dementia, etc). Further study should evaluate with various cognitive dysfunction. We wrote this limitation in discussion section [P16, L22-23 ~ P17, L1] as below:

“No dementia subtypes or MCI subtypes were specifically examined.”

- In Table 1, I would refer to the MMSE-2 and MoCA as cognitive screening tests rather than neuropsychological tests.

→ We are grateful for this insightful comment. As the reviewer’s comments, we revised the sub-title (neuropsychological tests -> cognitive screening tests) in table 1.

Altogether, my recommendation is to accept the manuscript pending minor revisions.

Attachment Submitted filename: 20210603.Response Reviewers_PLOS ONE.docx Click here for additional data file.

# Peer review: Decision Letter 1

**DOI:** 10.1371/journal.pone.0254055.r003

**Authors:** Antony Bayer

21 Jun 2021

Converting from the Montreal Cognitive Assessment to the Mini-Mental State Examination-2

PONE-D-21-11110R1

Dear Dr. PARK,

We’re pleased to inform you that your manuscript has been judged scientifically suitable for publication and will be formally accepted for publication once it meets all outstanding technical requirements.

Within one week, you’ll receive an e-mail detailing the required amendments. When these have been addressed, you’ll receive a formal acceptance letter and your manuscript will be scheduled for publication.

An invoice for payment will follow shortly after the formal acceptance. To ensure an efficient process, please log into Editorial Manager at http://www.editorialmanager.com/pone/, click the 'Update My Information' link at the top of the page, and double check that your user information is up-to-date. If you have any billing related questions, please contact our Author Billing department directly at authorbilling@plos.org.

If your institution or institutions have a press office, please notify them about your upcoming paper to help maximize its impact. If they’ll be preparing press materials, please inform our press team as soon as possible -- no later than 48 hours after receiving the formal acceptance. Your manuscript will remain under strict press embargo until 2 pm Eastern Time on the date of publication. For more information, please contact onepress@plos.org.

Kind regards,

Antony Bayer

Academic Editor

PLOS ONE

Additional Editor Comments (optional):

Reviewers' comments:

Reviewer's Responses to Questions

**Comments to the Author**

1. If the authors have adequately addressed your comments raised in a previous round of review and you feel that this manuscript is now acceptable for publication, you may indicate that here to bypass the “Comments to the Author” section, enter your conflict of interest statement in the “Confidential to Editor” section, and submit your "Accept" recommendation.

Reviewer #1: All comments have been addressed

Reviewer #2: All comments have been addressed

Reviewer #3: All comments have been addressed

**********

2. Is the manuscript technically sound, and do the data support the conclusions?

The manuscript must describe a technically sound piece of scientific research with data that supports the conclusions. Experiments must have been conducted rigorously, with appropriate controls, replication, and sample sizes. The conclusions must be drawn appropriately based on the data presented. 

Reviewer #1: Yes

Reviewer #2: Yes

Reviewer #3: Yes

**********

3. Has the statistical analysis been performed appropriately and rigorously? 

Reviewer #1: Yes

Reviewer #2: Yes

Reviewer #3: Yes

**********

4. Have the authors made all data underlying the findings in their manuscript fully available?

The PLOS Data policy requires authors to make all data underlying the findings described in their manuscript fully available without restriction, with rare exception (please refer to the Data Availability Statement in the manuscript PDF file). The data should be provided as part of the manuscript or its supporting information, or deposited to a public repository. For example, in addition to summary statistics, the data points behind means, medians and variance measures should be available. If there are restrictions on publicly sharing data—e.g. participant privacy or use of data from a third party—those must be specified.

Reviewer #1: No

Reviewer #2: Yes

Reviewer #3: Yes

**********

5. Is the manuscript presented in an intelligible fashion and written in standard English?

PLOS ONE does not copyedit accepted manuscripts, so the language in submitted articles must be clear, correct, and unambiguous. Any typographical or grammatical errors should be corrected at revision, so please note any specific errors here.

Reviewer #1: Yes

Reviewer #2: Yes

Reviewer #3: Yes

**********

6. Review Comments to the Author

Please use the space provided to explain your answers to the questions above. You may also include additional comments for the author, including concerns about dual publication, research ethics, or publication ethics. (Please upload your review as an attachment if it exceeds 20,000 characters)

Reviewer #1: (No Response)

Reviewer #2: All initial reviews address. I have no additional comments. This article should be accepted for publication.

Reviewer #3: My comments have been appropriately addressed. I have no further comment. I feel that the manuscript is ready for publication.

**********

7. PLOS authors have the option to publish the peer review history of their article (what does this mean?). If published, this will include your full peer review and any attached files.

If you choose “no”, your identity will remain anonymous but your review may still be made public.

**Do you want your identity to be public for this peer review?** For information about this choice, including consent withdrawal, please see our Privacy Policy.

Reviewer #1: **Yes: **AJ Larner

Reviewer #2: **Yes: **David Roalf

Reviewer #3: **Yes: **David Bergeron

# Editor report: Acceptance letter

**DOI:** 10.1371/journal.pone.0254055.r004

**Authors:** Antony Bayer

28 Jun 2021

PONE-D-21-11110R1 

Converting from the Montreal Cognitive Assessment to the Mini-Mental State Examination-2 

Dear Dr. PARK:

I'm pleased to inform you that your manuscript has been deemed suitable for publication in PLOS ONE. Congratulations! Your manuscript is now with our production department. 

If your institution or institutions have a press office, please let them know about your upcoming paper now to help maximize its impact. If they'll be preparing press materials, please inform our press team within the next 48 hours. Your manuscript will remain under strict press embargo until 2 pm Eastern Time on the date of publication. For more information please contact onepress@plos.org.

If we can help with anything else, please email us at plosone@plos.org. 

Thank you for submitting your work to PLOS ONE and supporting open access. 

Kind regards, 

PLOS ONE Editorial Office Staff

on behalf of

Professor Antony Bayer 

Academic Editor

PLOS ONE
